# Recent patents in allergy and immunology: A quantitative real‐time polymerase chain reaction method for diagnosing asthma and asthma exacerbation

**DOI:** 10.1002/clt2.12136

**Published:** 2022-03-31

**Authors:** Pureun‐Haneul Lee, SeonMuk Choi, MinHyeok An, An‐Soo Jang

**Affiliations:** ^1^ Division of Allergy and Respiratory Medicine, Department of Internal Medicine Soonchunhyang University Bucheon Hospital Bucheon Korea

**Keywords:** asthma, diagnosis, nectin‐4, qRT‐PCR

## Abstract

**Background:**

Asthma is diagnosed based on a history of the characteristic symptoms and evidence of expiratory airflow limitation. However, asthma diagnosis using the existing tests is associated with a risk of coronavirus disease 2019 caused by severe acute respiratory syndrome coronavirus 2 spread. In this study, we developed a quantitative real‐time polymerase chain reaction (qRT‐PCR)‐based asthma diagnosis tool.

**Methods:**

We detected nectin‐4 in the plasma of patients with asthma using qRT‐PCR, explored the relationship of clinical variables.

**Results:**

quantitative real‐time polymerase chain reaction revealed that plasma nectin‐4 mRNA levels were higher in asthmatics than controls. These results correlated with lung function.

**Conclusions:**

Those results suggest that qRT‐PCR for nectin‐4 may aid asthma diagnosis and monitoring.

1

Asthma is a heterogenous disease usually characterized by chronic airway inflammation and a history of respiratory symptoms, such as wheezing, shortness of breath, chest tightness, and cough, which vary in intensity over time. Expiratory airflow limitation^1^ is also seen in asthma, and may become persistent under the influence of the mucociliary escalator and secreted antimicrobial products, and when there is damage to intercellular protein junctions.^2^ Asthma is diagnosed based on a history of the characteristic symptoms and evidence of expiratory airflow limitation; the latter should be confirmed by bronchodilator reversibility testing, measurement of peak expiratory flow variability, an exercise or bronchial challenge test. Excessive variation in lung function between visits (which has specificity but low sensitivity), a significant improvement in lung function after 4 weeks of anti‐inflammatory treatment, and/or allergy tests and measurement of exhaled nitric oxide.^1^

Differential diagnosis of asthma varies by age; alternative diagnoses include chronic obstructive pulmonary disease, bronchiectasis, parenchymal lung disease, cardiac failure, and cystic fibrosis. Diagnosis may be aided by quantitative real‐time polymerase chain reaction (qRT‐PCR) amplification of a target nucleic acid in biological fluid. qRT‐PCR monitors amplification in real time, rather than only at the end of the reaction (unlike conventional PCR). qRT‐PCR products can be detected using non‐specific fluorescent dyes that intercalate with all double‐stranded DNAs or sequence‐specific complementary DNA oligonucleotides labeled with a fluorescent reporter.^3^

Recently, as coronavirus disease 2019^4^ caused by severe acute respiratory syndrome coronavirus 2 (SARS‐CoV‐2) spread rapidly worldwide, quick and accurate examination and diagnosis became imperative. Commercial PCR assays have been approved by the US Food and Drug Administration (FDA) for emergency detection of SARS‐CoV‐2 nucleic acid in nasopharyngeal, oropharyngeal, and anterior/mid‐turbinate nasal swabs, nasopharyngeal aspirates, bronchoalveolar lavage fluid, and saliva.^4^ Asthma diagnosis using the existing tests is associated with a risk of SARS‐CoV‐2 spread. Thus, we developed a qRT‐PCR‐based asthma diagnosis tool.

The basic principle (Figure 1A) of our tool is that the pulmonary airway epithelium is a critical external interface often exposed to harmful aerosols and pathogens.^5^ The proximal bronchial epithelium has columnar ciliated cells and mucus‐secreting goblet cells, which are supported by basal cells. Together, they inhibit fluid loss, pathogen entry, and inappropriate immune reactions in the subepithelial lung mucosa.^5^ Nectin‐4 (a protein of the epithelial adherens junction) plays important roles in both acquired immunity and angiogenesis, suggesting that it might also exert other functions.^5^ Cell degradation, proteolysis, and alternative splicing release soluble nectin‐4 into cell supernatants.^5^

**FIGURE 1:**
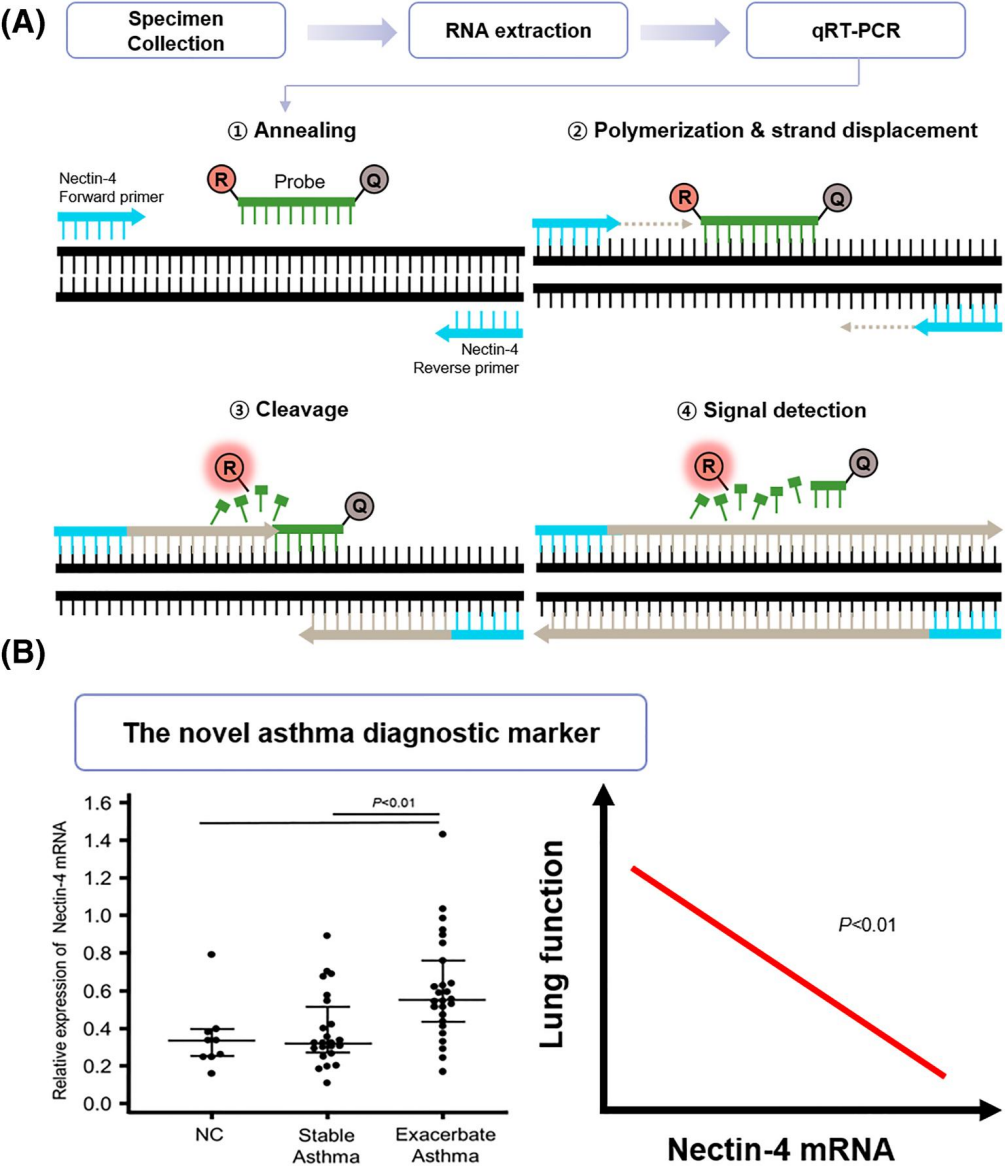
(A) Overview of the nectin‐4 quantitative real‐time polymerase chain reaction assay, (B) nectin‐4 mRNA levels in control subjects and asthma patients, and the correlation thereof with lung function

Nectin‐4 could serve as a novel diagnostic marker of asthma.^6^ qRT‐PCR revealed that plasma nectin‐4 mRNA levels were higher in asthmatics than controls; this may aid asthma diagnosis and monitoring (Figure 1B). However, the sensitivity and specificity of nectin‐4 require further assessment and comparison with those of recognized asthma tests.

## CONFLICT OF INTEREST

The authors declare that they have no competing interest.

## AUTHOR CONTRIBUTIONS

An‐Soo Jang designed the study and wrote the manuscript. Pureun‐Haneul Lee performed data collection, interpretation and journal submission assistance for this manuscript. SeonMuk Choi and MinHyeok An contributed to the conduct of the study and data collection. All authors read and approved the final manuscript.
